# Comparison of performance on a heat tolerance test of military personnel that have suffered heat illness and subsequently been diagnosed with malignant hyperthermia and controls

**DOI:** 10.1186/2046-7648-4-S1-A22

**Published:** 2015-09-14

**Authors:** Carol House, Dan Roiz de Sa

**Affiliations:** 1 Environmental Medicine and Science, Institute of Naval Medicine, Gosport, UK

## Introduction

The Institute of Naval Medicine runs a Heat Illness Clinic (HIC), seeing about 140 military patients a year who have been referred following an episode of heat illness (HI). Patients undertake assessment of maximal aerobic fitness (VO_2_max) followed by a heat tolerance test (HTT), in which they walk on a treadmill at 60 % VO_2_max in a warm environment (34 °C dry bulb, relative humidity 40 % with a WBGT of 27 °C). Initially patients are fully clothed and carry a rucksack (mass 14 kg) to raise the deep body temperature, at 30 min the jacket and rucksack are removed and at 45 min the t-shirt removed. Patients continue to exercise until thermal equilibrium (*i.e*. a plateau of rectal temperature) is achieved (60 min to 90 min). Rectal temperature (T_re_), mean skin temperature (T_msk_), heart rate (HR) and sweat rate (SR) are recorded. Patients in which thermal equilibrium is not achieved are considered to demonstrate abnormal thermoregulation and "fail" the HTT. These patients are reviewed at least once a minimum of 8 weeks later, and if they consistently fail the HTT and there is a suspicion of malignant hyperthermia (MH) they are recommended for referral to the Malignant Hyperthermia Unit, Leeds. MH is a genetic condition in which too much calcium is released from the intracellular store into the cytoplasm in response to certain triggering agents, *e.g*. the anaesthetic halothane. This leads to a rise in cellular metabolism and heat production, particularly within skeletal muscle, and it has been suggested that individuals with underlying MH are more susceptible to HI [1].

## Methods

HIC data from patients that attended the clinic in 2014 and passed the HTT (n = 116), data from patients that had attended the HIC and were subsequently diagnosed with MH (n = 11), and data from military personnel with no history of HI who volunteered to undertake HIC assessments (controls, n = 19) were compared. Data were compared by one-way analysis of variance with pair-wise comparisons and Bonferroni correction using SPSS version 21, p < 0.05 was considered significant.

## Results

The MH group had a higher HR (45-60 min), T_re _at 60 min and greater rise of T_re _between 30 to 45 min and 45 to 60 min than the HIC patients that passed the HTT and the control group, and a lower absolute VO_2_max and higher T_msk _at 30, 45 and 60 min than HIC patients that passed the HTT. There were no differences in age, height, body mass, VO_2_max relative to body mass or SR (absolute or compared to body surface area) between the MH group and the other two groups.

## Discussion

The results suggest that individuals with MH will demonstrate heat intolerance when assessed in the INM HIC, and this may make them more susceptible to HI. However, further work is required to determine the sensitivity and specificity of the HTT to detect MH.

## Conclusion

Individuals with MH may demonstrate heat intolerance, which can be identified using the INM HTT.

## References

[B1] Hopkins PM Is there a link between malignant hyperthermia and exertional heat illness? British Journal of Sports Medicine 2007 41 283 284 10.1136/bjsm.2006.032516 17261558 PMC2659049

